# Supplementary figures and images for: The Legionella pneumophila type IVb secretion system effector BinA subverts amino acid transport to sensitize TORC1 signaling in macrophages
Source: PLoS Pathog. 2026 Jun 8;22(6):e1012998. doi: 10.1371/journal.ppat.1012998 (PMC13258155; doi:10.1371/journal.ppat.1012998)

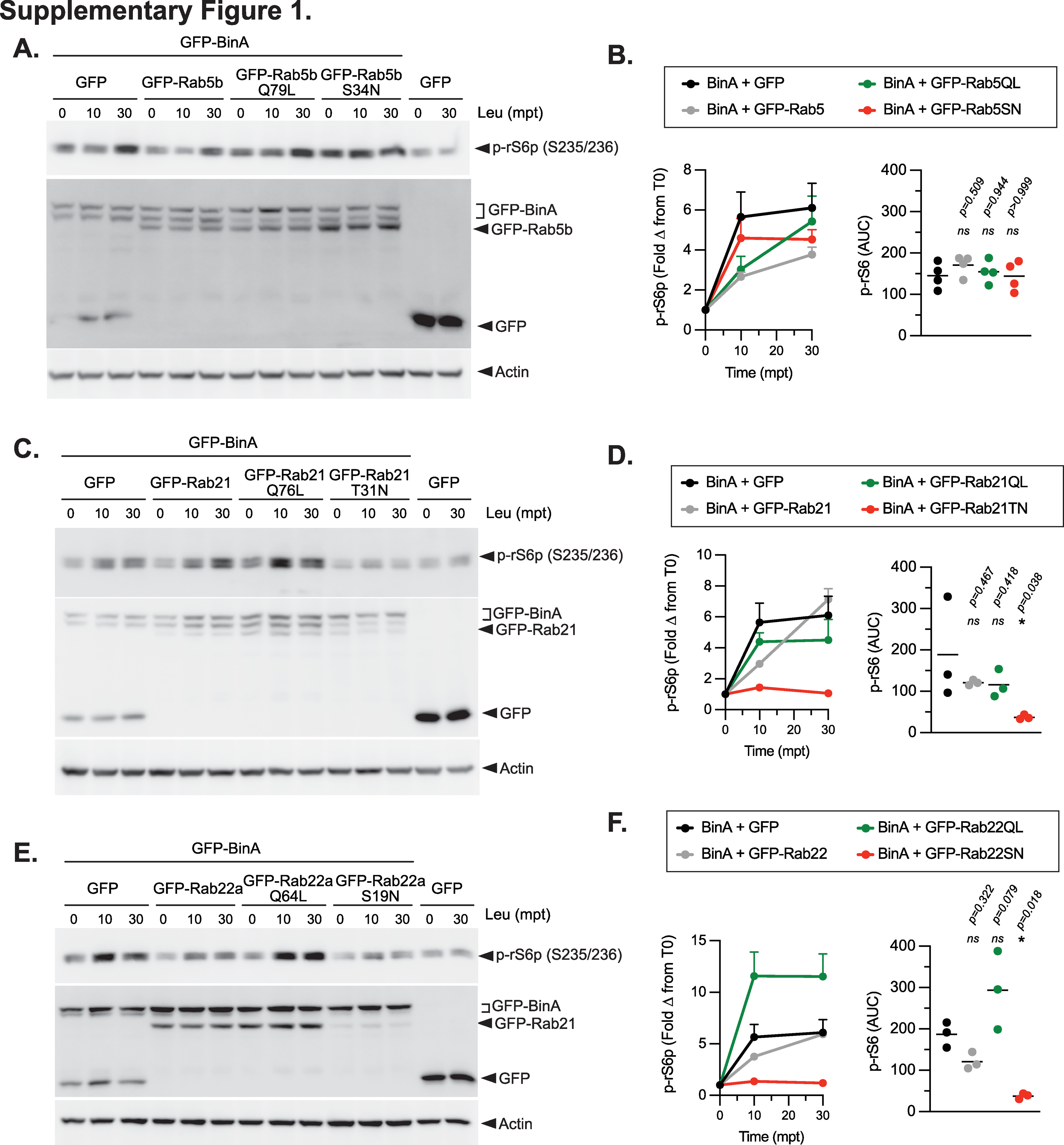

Supplement: S1 Fig — Kinetics of TORC1 activation triggered by starvation/refeeding stimulation with 100µM Leu in HEK293 cells producing BinA in the presence or absence of different Rab5 (A-B), Rab21 (C-D), or Rab22a (E-F) alleles. (B, D and F) Quantitative analyses of band signal intensities show Averages ± StDev from at least three biological repeats (left panels) and the respective area-under-the curve (AUC) analyses are presented in the right panels. Statistical analyses were completed with one-way ANOVA with Dunnett’s multiple comparison test using the ‘BinA+GFP’ as control group and p-values are indicated in the respective data panels. (TIF) [file ppat.1012998.s001.tif]

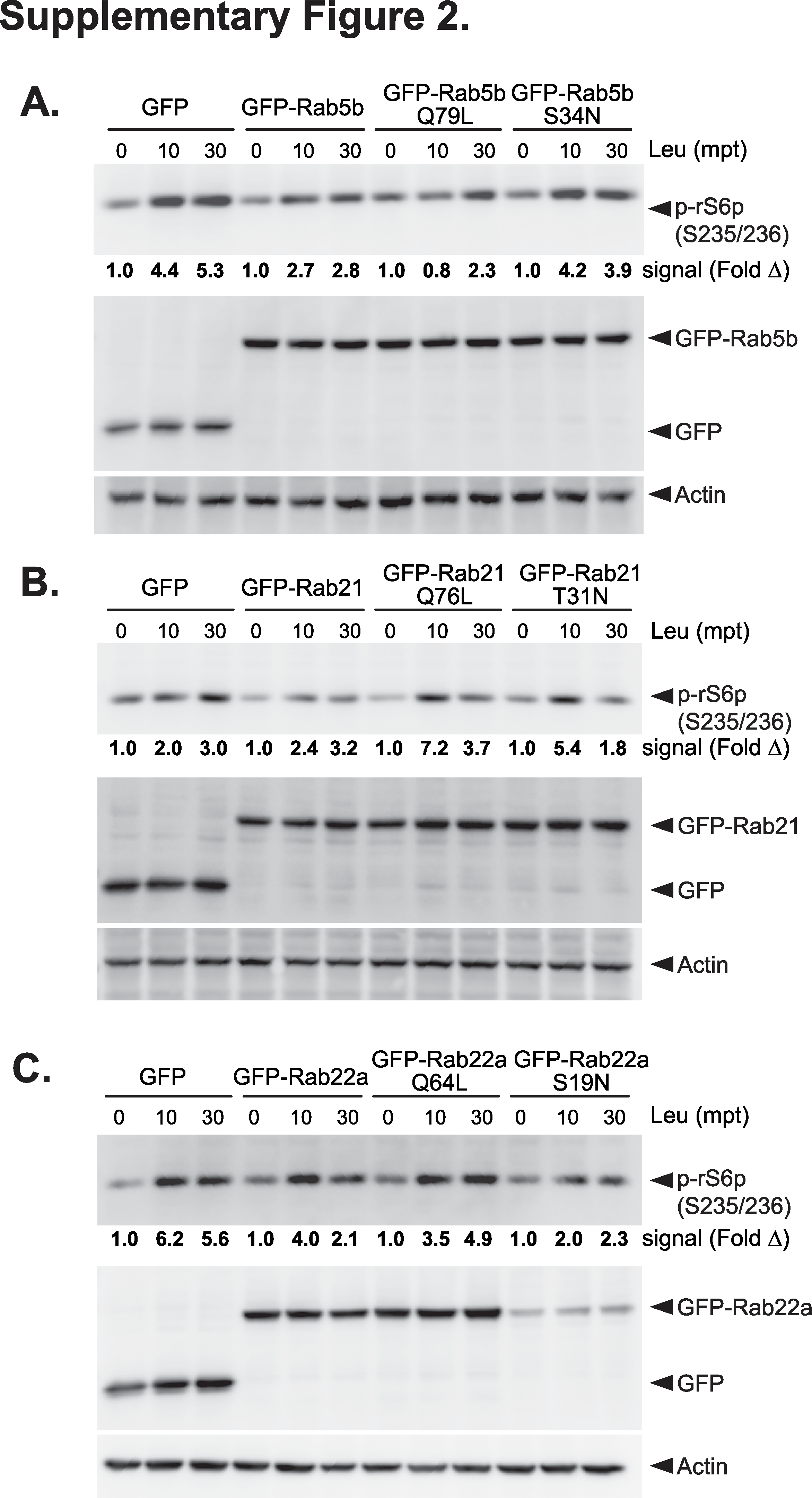

Supplement: S2 Fig — Kinetics of TORC1 activation triggered by starvation/refeeding stimulation with 400µM Leu in HEK293 cells producing GFP or different Rab5 (A), Rab21 (B), or Rab22a (C) alleles. Band signal intensity in the phospho-immunoblot for rS6p for each condition was quantified and is presented below the respective Fig panel as fold change from untreated cells. The data shown is from one experiment out of three biological replicates. (TIF) [file ppat.1012998.s002.tif]

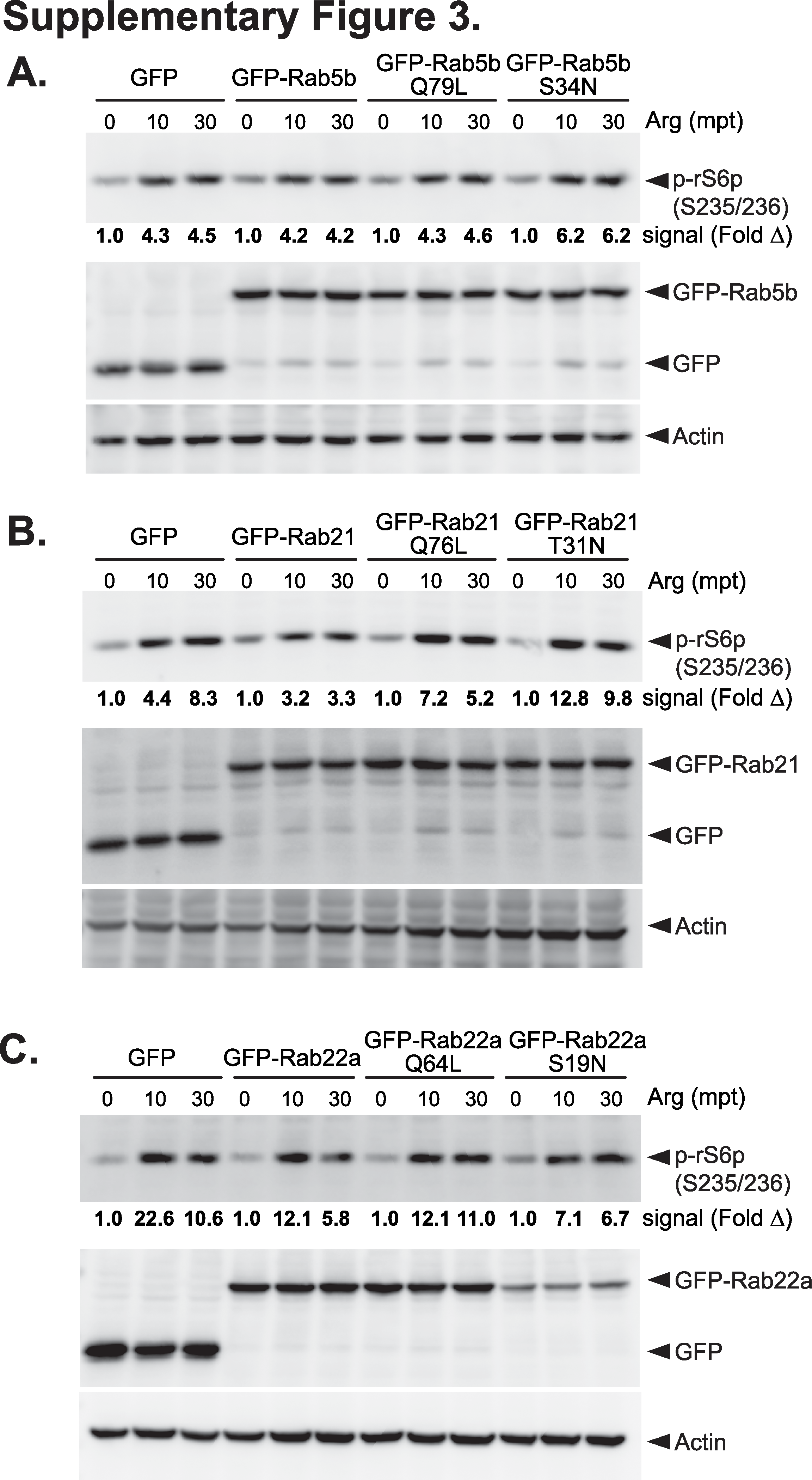

Supplement: S3 Fig — Kinetics of TORC1 activation triggered by starvation/refeeding stimulation with 1mM Arg in HEK293 cells producing GFP or different Rab5 (A), Rab21 (B), or Rab22a (C) alleles. Band signal intensity in the phospho-immunoblot for rS6p for each condition was quantified and is presented below the respective Fig panel as fold change from untreated cells. The data shown is from one experiment out of three biological replicates. (TIF) [file ppat.1012998.s003.tif]

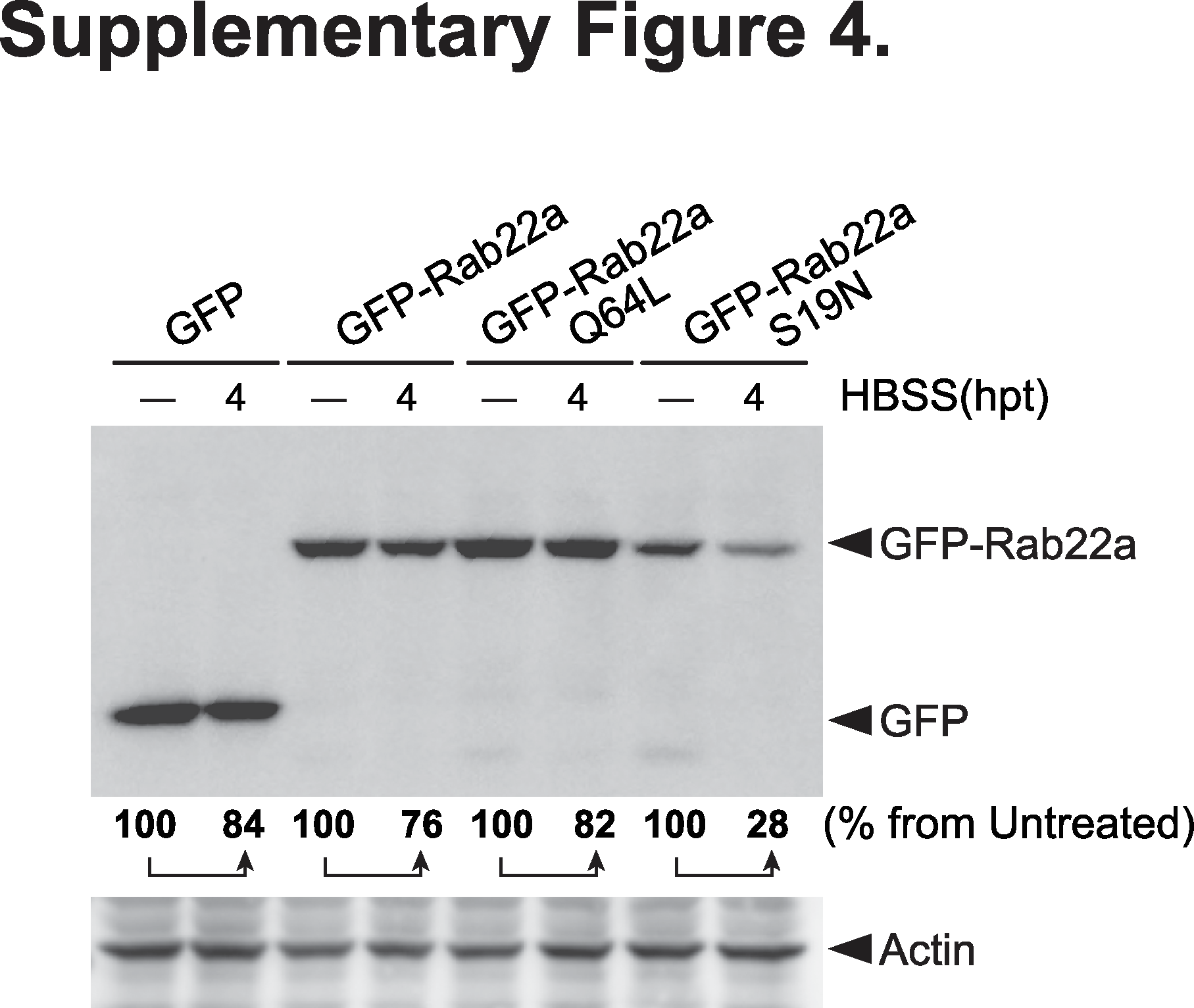

Supplement: S4 Fig — HEK293 cells ectopically producing GFP alone or the indicated GFP-tagged Rab22a alleles were treated with HBSS for 4 hours. Immunoblot analysis demonstrates reduction of total Rab22a S19N abundance upon amino acid withdrawal. Band signal intensity for GFP blots for each condition was quantified and is presented below the respective Fig panels as percentage of signal from cells that were not treated with HBSS. The data shown is from one experiment out of three biological replicates. (TIF) [file ppat.1012998.s004.tif]

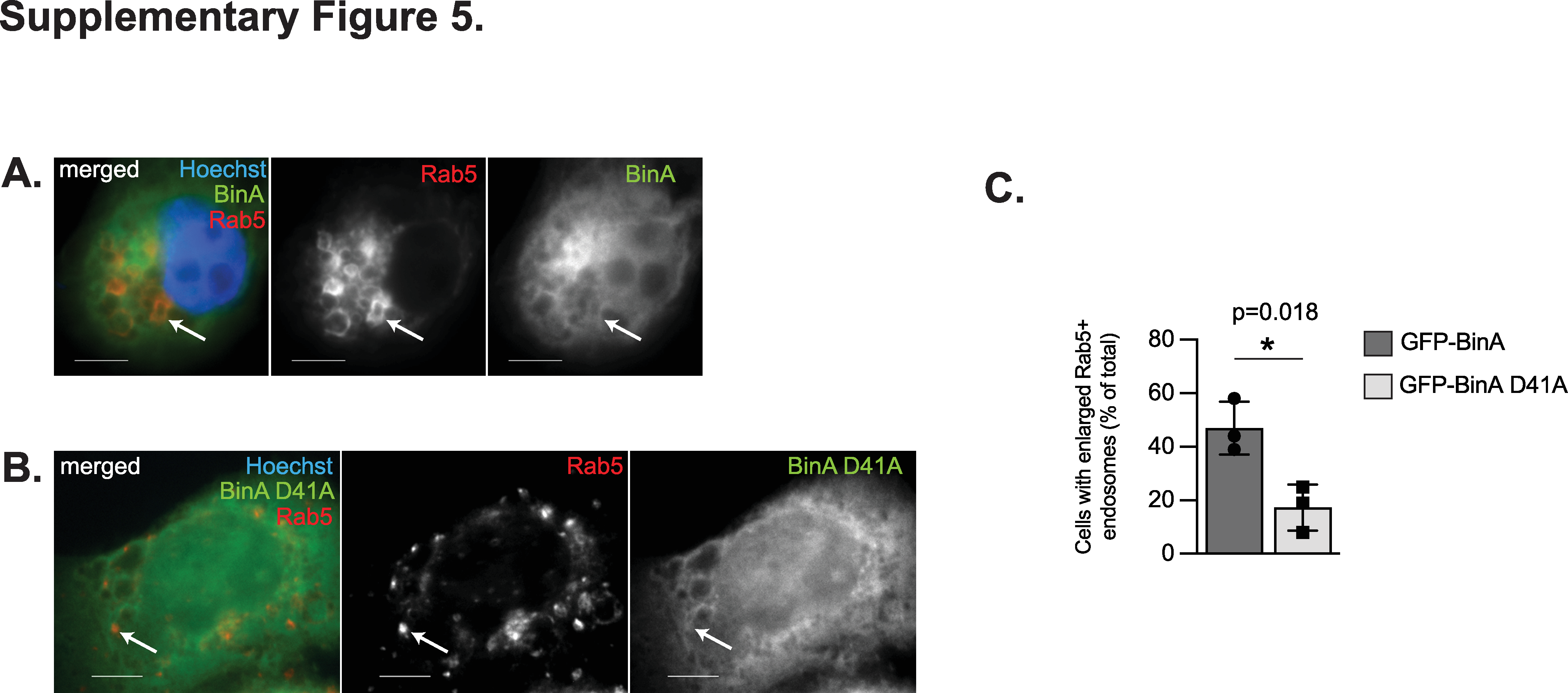

Supplement: S5 Fig — (A-B) Representative micrographs of A549 cells co-expressing RFP-tagged Rab5 and either GFP-BinA (A) or GFP-BinA D41A (B). Merged and the respective single channel images are shown. (C) Quantitative analysis of large Rab5 vesicles produced by BinA and BinA D41A-expressing cells. The graph shows Averages ± StDev from three biological replicates where at least 100 cells were counted for each condition. Statistical analysis was completed with unpaired T-test with Welch’s correction and the p-value is indicated in the graph panel. (TIF) [file ppat.1012998.s005.tif]
