# Supplementary material for: The Legionella pneumophila type IVb secretion system effector BinA subverts amino acid transport to sensitize TORC1 signaling in macrophages
Source: PLoS Pathog. 2026 Jun 8;22(6):e1012998. doi: 10.1371/journal.ppat.1012998 (PMC13258155; doi:10.1371/journal.ppat.1012998)
Supplement: S1 Table — List of all transposon mutants included in the forward genetic screen and their respective z-scores. (PDF) [file ppat.1012998.s006.pdf]

Supplementary Table 1. Forward genetic Tm-based screen for bacterial TORC1 regulators.

	Transposon insertion in ORF	Gene name	BMDMs containing only Ub+ LCVs (z-score)	All infected BMDMs (z-score)
**1**	lpg0012	*cegC1*	-0.04	1.09
**2**	lpg0030	*ravB*	0.36	-0.62
**3**	lpg0038	*legA10/ankQ*	-0.91	-0.93
**4**	lpg0045		0.12	-0.40
**5**	lpg0059	*ceg2*	1.58	1.48
**6**	lpg0086		-0.67	-0.92
**7**	lpg0090	*lem1*	0.50	1.18
**8**	lpg0103	*vipF*	1.06	1.09
**9**	lpg0135	*sdhB*	1.56	-2.10
**10**	lpg0140		-0.92	-0.27
**11**	lpg0160	*ravD*	0.92	1.32
**12**	lpg0171	*legU1*	-0.51	-0.97
**13**	lpg0172		-0.45	0.80
**14**	lpg0195	*ravE*	-1.25	0.20
**15**	lpg0196	*ravF*	0.66	0.76
**16**	lpg0208	*ceg6*	-0.17	-0.49
**17**	lpg0209	*mavR*	-2.23	-1.64
**18**	lpg0210	*ravG*	-2.48	-1.23
**19**	lpg0234	*sidE*	0.50	-0.25
**20**	lpg0240	*ceg8*	-1.16	-0.07
**21**	lpg0246	*ceg9*	0.21	1.19
**22**	lpg0254		0.12	0.56
**23**	lpg0275	*sdbA*	-1.46	-0.66
**24**	lpg0284	*ceg10*	-1.08	-0.24
**25**	lpg0285	*lem2*	0.61	-0.01
**26**	lpg0365		-0.19	-1.19
**27**	lpg0376	*sdhA*	-0.30	-2.05
**28**	lpg0393	***binA***	**-3.90**	**-2.80**
**29**	lpg0401	*ceg11*	0.05	-0.86
**30**	lpg0402	*legA9/ceg12/ankY*	0.50	0.69
**31**	lpg0403	*legA7/ankG/ankZ*	-1.78	-1.15
**32**	lpg0405		0.14	0.19
**33**	lpg0436	*legA11/ankJ*	-0.58	-1.68
**34**	lpg0437	*ceg14*	-0.17	1.02
**35**	lpg0483	*legA12/ankC*	-0.22	0.42
**36**	lpg0515	*legD2*	1.56	1.47
**37**	lpg0518		0.25	-0.21
**38**	lpg0563		-0.65	-0.05

**39**	lpg0621	*sidA*	-0.11	0.92
**40**	lpg0634		0.86	0.64
**41**	lpg0642	*wipB*	-0.36	0.99
**42**	lpg0645	*mavS*	1.56	0.62
**43**	lpg0695	*legA8/ankN/ankX*	-0.09	1.78
**44**	lpg0696	*lem3*	1.03	0.33
**45**	lpg0716		-0.62	-0.09
**46**	lpg0733	*ravH*	-0.39	0.04
**47**	lpg0796		-0.11	0.47
**48**	lpg0921	*mavT*	-0.54	0.08
**49**	lpg0926	*ravI*	-0.54	-0.34
**50**	lpg0944	*ravJ*	-0.92	-1.28
**51**	lpg0967		1.58	1.25
**52**	lpg0968	*sidK*	0.50	-0.57
**53**	lpg1101	*lem4*	-1.50	-1.68
**54**	lpg1108	*ravL*	-1.14	-0.60
**55**	lpg1121	*ceg19*	0.40	-1.36
**56**	lpg1137		0.72	0.73
**57**	lpg1144	*cegC3*	1.70	2.34
**58**	lpg1145	*lem7*	1.05	-0.78
**59**	lpg1227	*vpdB*	1.04	0.31
**60**	lpg1273		0.77	0.32
**61**	lpg1290	*lem8*	1.58	-0.39
**62**	lpg1312	*legC1*	-0.51	0.06
**63**	lpg1316	*ravT*	0.94	-0.35
**64**	lpg1317	*ravW*	-0.51	-0.36
**65**	lpg1354	*sidV*	-0.30	0.26
**66**	lpg1355	*sidG*	1.14	1.37
**67**	lpg1356		-0.30	0.47
**68**	lpg1408	*licA*	0.78	1.54
**69**	lpg1449		0.19	-0.32
**70**	lpg1453		0.50	-0.16
**71**	lpg1488	*legC5*	0.31	-0.34
**72**	lpg1489	*ravX*	-0.25	0.94
**73**	lpg1491	*lem9*	1.11	-0.31
**74**	lpg1496	*lem10*	-0.17	-1.52
**75**	lpg1551	*ravY*	1.70	1.61
**76**	lpg1588	*legC6*	1.58	-1.01
**77**	lpg1625	*lem12*	-0.15	0.33
**78**	lpg1642	*sidB*	1.14	-0.40
**79**	lpg1654		-1.53	-1.59
**80**	lpg1660	*legL3*	-0.77	0.34

**81**	lpg1663		-1.06	-0.25
**82**	lpg1685		-0.65	-0.27
**83**	lpg1692		0.81	-0.28
**84**	lpg1701	*legC3*	0.57	0.63
**85**	lpg1702	*lem13*	1.15	1.33
**86**	lpg1716		0.36	1.19
**87**	lpg1718	*legAS4/ankI*	-0.37	-1.46
**88**	lpg1751		0.88	1.33
**89**	lpg1776		-0.08	-1.02
**90**	lpg1798	*mavU*	1.03	-0.06
**91**	lpg1822		1.09	2.48
**92**	lpg1884	*legC2/ylfB*	0.12	1.10
**93**	lpg1888		-0.11	-0.96
**94**	lpg1890	*legLC8*	0.97	-1.03
**95**	lpg1930		0.92	0.87
**96**	lpg1947	*lem16*	0.14	-1.15
**97**	lpg1948	*legLC4*	-0.52	0.39
**98**	lpg1953	*legC4*	-3.01	-1.18
**99**	lpg1958	*legL5*	-0.75	-1.32
**100**	lpg1959		0.63	0.23
**101**	lpg1961		-0.86	-0.89
**102**	lpg1962	*lirB*	-0.44	0.53
**103**	lpg1963	*pieA/lirC*	0.94	0.29
**104**	lpg1964	*lirD*	-0.36	0.32
**105**	lpg1965	*lirE*	-1.06	-1.15
**106**	lpg1966	*lirF*	-0.15	-0.48
**107**	lpg1972	*pieF*	-0.56	-0.08
**108**	lpg2050		1.02	-0.04
**109**	lpg2131	*legA6*	0.63	0.58
**110**	lpg2137	*legK2*	-0.25	-0.33
**111**	lpg2144	*legAU13/ceg27/ankB*	0.31	0.25
**112**	lpg2147	*mavC*	-0.86	-1.78
**113**	lpg2148		-2.26	-0.81
**114**	lpg2155	*sidJ*	1.56	1.87
**115**	lpg2156	*sdeB*	-0.15	-0.81
**116**	lpg2176	*legS2*	0.25	0.49
**117**	lpg2199	*mavD*	-1.13	0.79
**118**	lpg2222		1.58	1.53
**119**	lpg2223		-1.06	-1.04
**120**	lpg2224	*ppgA*	-0.91	-0.57
**121**	lpg2239		-1.16	-1.56
**122**	lpg2244		-1.23	0.77

**123**	lpg2248	*lem21*	0.85	0.09
**124**	lpg2283		0.36	-0.19
**125**	lpg2300	*legA3/ankH/ankW*	-0.09	-0.55
**126**	lpg2311	*ceg28*	1.56	-1.09
**127**	lpg2322	*legA5/ankK*	-3.08	-1.88
**128**	lpg2327	*ceg6*	-2.07	-1.98
**129**	lpg2328	*lem22*	-0.04	0.12
**130**	lpg2375		0.07	-1.09
**131**	lpg2391	*sdbC*	0.73	1.85
**132**	lpg2395		0.32	0.85
**133**	lpg2400	*legL7*	-1.06	-0.41
**134**	lpg2407		-0.09	-0.41
**135**	lpg2409	*ceg29*	0.72	0.62
**136**	lpg2416	*legA1*	0.82	-1.71
**137**	lpg2420		0.42	1.47
**138**	lpg2424	*mavG*	0.47	0.62
**139**	lpg2425	*mavH*	0.50	-0.03
**140**	lpg2433	*ceg30*	0.88	-0.37
**141**	lpg2456	*legA15/ankD*	1.29	1.48
**142**	lpg2482	*sdbB*	-1.14	-0.50
**143**	lpg2490	*lepB*	0.66	-0.27
**144**	lpg2498	*mavJ*	-0.30	0.33
**145**	lpg2508	*sdjA*	-1.06	-0.78
**146**	lpg2509	*sdeD*	0.99	1.23
**147**	lpg2510	*sdcA*	-1.62	-0.81
**148**	lpg2511	*sidC*	1.03	1.17
**149**	lpg2526	*mavL*	1.10	1.37
**150**	lpg2529	*lem27*	-1.02	0.01
**151**	lpg2539		0.14	-0.81
**152**	lpg2584	*sidF*	-1.86	-1.23
**153**	lpg2588	*legS1*	-0.50	-0.39
**154**	lpg2591	*ceg33*	0.67	1.04
**155**	lpg2603	*sdmB/lem28*	0.66	1.09
**156**	lpg2628		-0.86	-0.36
**157**	lpg2637		-1.25	-0.74
**158**	lpg2694	*legD1*	1.70	2.43
**159**	lpg2718	*wipA*	0.25	0.48
**160**	lpg2744		0.72	1.52
**161**	lpg2793	*lepA*	0.72	1.41
**162**	lpg2804	*lem29*	1.10	1.23
**163**	lpg2815	*mavN*	0.99	0.38
**164**	lpg2819		1.10	1.41

**165**	lpg2826	*ceg34*	-0.09	0.49
**166**	lpg2829	*sidH*	-0.25	-1.41
**167**	lpg2830	*legU2/lubX*	0.69	0.44
**168**	lpg2831	*vipD*	0.57	0.28
**169**	lpg2844		1.09	0.01
**170**	lpg2862	*legC8*	-0.96	-0.32
**171**	lpg2874		0.92	2.01
**172**	lpg2879	*mavO*	0.17	0.01
**173**	lpg2885		0.25	0.85
**174**	lpg2912		-0.67	0.14
**175**	lpg2975	*mavQ*	0.40	0.78
**176**	lpg2999	*legP*	1.58	-0.36
**177**	lpg3000		0.07	0.20
