# Supplementary material for: The Legionella pneumophila type IVb secretion system effector BinA subverts amino acid transport to sensitize TORC1 signaling in macrophages
Source: PLoS Pathog. 2026 Jun 8;22(6):e1012998. doi: 10.1371/journal.ppat.1012998 (PMC13258155; doi:10.1371/journal.ppat.1012998)
Supplement: S2 Table — (PDF) [file ppat.1012998.s007.pdf]

Supplementary Table 2. Strains used in this study

Strains	Genotype	Properties	Reference
CR19	*E. coli* DH5α λpir *endA1 hsdR17 glnV44 (= supE44) thi-1 recA1 gyrA96 relA1* φ80d*lac*Δ*(lacZ)M15* Δ*(lacZYA-argF)U169 zdg-232*::Tn10 *uidA*::pir^{+}	conjugation helper strain	[86]
Lp01 Δ*flaA*	*L. pneumophila* Lp01 *ΔflaA*	*flaA* clean deletion mutant	[4]
Lp01 Δ*flaA* Δ*binA*	*L. pneumophila* Lp01 *ΔflaA* Δ*binA*	*flaA binA* clean deletion mutant	This study
Lp01 Δ*flaA* Δ*binA* + EV	*L. pneumophila* Lp01 *ΔflaA* Δ*binA* pJB1806-3xFlag	*flaA binA* mutant complemented with empty pJB1806 plasmid	This study
Lp01 Δ*flaA* Δ*binA* + pBinA	*L. pneumophila* Lp01 *ΔflaA* Δ*binA* pJB1806-3xFlag-BinA	*flaA binA* mutant complemented with 3XFlag-tagged BinA under an IPTG-inducible promoter	This study
Lp01 Δ*flaA* Δ*binA* + pBinA D41A	*L. pneumophila* Lp01 *ΔflaA* Δ*binA* pJB1806-3xFlag-BinA D41A	*flaA binA* mutant complemented with 3XFlag-tagged BinA D41A under an IPTG-inducible promoter	This study
Lp01 Δ*flaA* GFP+	*L. pneumophila* LP01 Δ*flaA p*Ptac-GFP	inducible GFP expression, plasmid-borne pAM239	[43]
Lp01 Δ*flaA* Δ*binA* GFP+	*L. pneumophila* LP01 Δ*flaA* Δ*binA p*Ptac-GFP	inducible GFP expression, plasmid-borne pAM239	This study
