# Supplementary material for: The Legionella pneumophila type IVb secretion system effector BinA subverts amino acid transport to sensitize TORC1 signaling in macrophages
Source: PLoS Pathog. 2026 Jun 8;22(6):e1012998. doi: 10.1371/journal.ppat.1012998 (PMC13258155; doi:10.1371/journal.ppat.1012998)
Supplement: S3 Table — (PDF) [file ppat.1012998.s008.pdf]

Supplementary Table 3. Primers used in this study

Designation	Primer sequence (5'→ 3')	RE site
F_BamHI_+2_lpg0393-*binA*	CGGGATCCTTCTTTCATCAACAAAGGAATATCTTCAAGC	BamHI
R_SbFI_lpg0393-*binA*	GCCCTGCAGGTTAAACTTTGGGATTCTTATCCGTATCAG	SbFI
F_BamHI+2_lpg0393_(250nt)	CGGGATCCTTTATGCACTCACTACAATTTCTATCGC	BamHI
F_BamHI+2_lpg0393_(304nt)	CGGGATCCTTAATAATCTGCAAGAGCATTATATTACTTC	BamHI
F_BamHI+2_lpg0393_(385nt)	CGGGATCCTTTATCTTGAGAAGCAACGGTTTGTCG	BamHI
R_SbFI_lpg0393_(390nt)	GCCCTGCAGGTTAAAGATAAGCATTGTTCATAGTCATTAA TTGG	SbFI
R_SbFI_lpg0393_(510nt)	GCCCTGCAGGTTAATAGGTTACAGAATAAATTTGATCAAA CGC	SbFI
R_SbFI_lpg0393_(651nt)	GCCCTGCAGGTTAGACATCTTGAGTTAATTCGGTTTGCTC	SbFI
QC_F_lpg0393_D41A	CAAAAATCAAGAAGCTGCTGAAATGGTGAGCTTG	
QC_R_lpg0393_D41A	CAAGCTCACCATTTCAGCAGCTTCTTGATTTTTG	
QC_FSP_lpg0393_D41A	CAAAAATCAAGAAGCTGC	
UpF_CD_BamHI_*binA*	GTGGATCCTTACCTTGCAAATGTACTGGC	BamHI
UpR_CD_EcoRI_*binA*	CGGAATTCATAGTTATCCATTACCAAAATCTG	EcoRI
DownF_CD_EcoRI_*binA*	CGGAATTCTCTCACACGACTTGCTAATC	EcoRI
DownR_CD_SacI_*binA*	ATCGAGCTCCCAATTTGAGGAGTAGTCC	SacI
SP1_CD_*binA*	TAGGATAACAATTGATGTGCG	
SP2_CD_*binA*	AAGGCTTACTTCGACAATCC	
SP3_CD_*binA*	ATGAACGGCATACAAAATAGC	
