# Supplementary material for: The Legionella pneumophila type IVb secretion system effector BinA subverts amino acid transport to sensitize TORC1 signaling in macrophages
Source: PLoS Pathog. 2026 Jun 8;22(6):e1012998. doi: 10.1371/journal.ppat.1012998 (PMC13258155; doi:10.1371/journal.ppat.1012998)
Supplement: S4 Table — (PDF) [file ppat.1012998.s009.pdf]

Supplementary Table 4. Plasmids used in this study

Plasmids	Important properties	Marker	Reference
pSR47s	*oriT* mobRP4 R6K *ori*, *sacB* Kan^R - suicide gene replacement plasmid for allelic exchange	Kan	[86]
pSR47s-CDLpg0393	pSR47s containing the Δ*binA* clean deletion allele	Kan	This study
pJB1806-3XFlag	Bacterial plasmid for generation of 3XFlag-tagged gene alleles under the control of the IPTG-inducible *p*Ptac promoter, Cm^R, Amp^R	Amp/ Cm	[44]
pJB1806-3XFlag-BinA	IPTG-inducible expression of 3XFlag-tagged BinA	Amp/ Cm	This study
pJB1806-3XFlag-BinA D41A	IPTG-inducible expression of 3XFlag-tagged BinA D41A	Amp/ Cm	This study
pAM239	GFP under the control of the IPTG-inducible *p*Ptac promoter, Cm^R	Cm	[43]
pEGFP-C2	Eukaryotic expression plasmid for production of GFP fusion alleles	Kan	Clontech
pEGFP-BinA	Eukaryotic expression of GFP-BinA fusion allele	Kan	This study
pEGFP-BinA D41A	Eukaryotic expression of GFP-BinA D41A fusion allele	Kan	This study
pLB-GFP-BinA	Eukaryotic expression of GFP-BinA fusion allele	Amp	This study
pLB-GFP-BinA(84-287)	Eukaryotic expression of GFP-BinA fusion allele	Amp	This study
pLB-GFP-BinA(102-287)	Eukaryotic expression of GFP-BinA fusion allele	Amp	This study
pLB-GFP-BinA(129-287)	Eukaryotic expression of GFP-BinA fusion allele	Amp	This study
pLB-GFP-BinA(1-130)	Eukaryotic expression of GFP-BinA fusion allele	Amp	This study
pLB-GFP-BinA(1-170)	Eukaryotic expression of GFP-BinA fusion allele	Amp	This study
pLB-GFP-BinA(1-217)	Eukaryotic expression of GFP-BinA fusion allele	Amp	This study
pLB-GFP-mRab21	Eukaryotic expression of the fusion allele GFP-mouse Rab21	Amp	This study
pLB-GFP-mRab21 T31N<br>pLB-GFP-hRab22a	Eukaryotic expression of the fusion allele GFP-mouse Rab21 T31N	Amp	This study
 pLB-GFP-hRagA Q66L	Eukaryotic expression of the fusion allele GFP-human Rab22a	Amp	This study
	Eukaryotic expression of the fusion allele GFP-human RagA Q66L	Amp	This study
pEGFP-hRab5b	Eukaryotic expression of the fusion allele GFP-human Rab5b	Kan	This study
pEGFP-hRab5b Q79L	Eukaryotic expression of the fusion allele GFP-human Rab5b Q79L	Kan	This study
pEGFP-hRab5b S34N	Eukaryotic expression of the fusion allele GFP-human Rab5b S34N	Kan	This study
pEGFP-hRab22a	Eukaryotic expression of the fusion allele GFP-human Rab22a	Kan	This study
pEGFP-hRab22a Q64L	Eukaryotic expression of the fusion allele GFP-human Rab22a Q64L	Kan	This study
pEGFP-hRab22a S19N	Eukaryotic expression of the fusion allele GFP-human Rab22a S19N	Kan	This study
pEGFP-hRagA Q66L	Eukaryotic expression of the fusion allele GFP-human RagA Q66L	Kan	This study
pEGFP-hRagA T21N	Eukaryotic expression of the fusion allele GFP-human RagA T21N	Kan	This study
pEGFP-mRab21	Eukaryotic expression of the fusion allele GFP-mouse Rab21	Kan	Addgene, plasmid # 83421 [83]

pEGFP-mRab21 Q76L	Eukaryotic expression of the fusion allele GFP-mouse Rab21 Q76L	Kan	Addgene, plasmid # 83422 [83]
pEGFP-mRab21 T31N	Eukaryotic expression of the fusion allele GFP-mouse Rab21 T31N	Kan	Addgene, plasmid # 83423 [83]
