# Supplementary material for: The Legionella pneumophila type IVb secretion system effector BinA subverts amino acid transport to sensitize TORC1 signaling in macrophages
Source: PLoS Pathog. 2026 Jun 8;22(6):e1012998. doi: 10.1371/journal.ppat.1012998 (PMC13258155; doi:10.1371/journal.ppat.1012998)
Supplement: Attachment — Submitted filename: Response to Reviewers.docx [file ppat.1012998.s011.docx]

Apr 15th, 2026

**Responses to reviewers’ comments and critiques:**

We would like to express our gratitude for the positive feedback and the insightful critiques of all the reviewers. The novelty and rigor of our work was noted by all reviewers, however two reviewers (R1 and R3) felt that further insight into BinA mechanism is needed. In the revision, we provide additional data that further illuminates the mechanism of BinA-driven TORC1 sensitization. First, we show that while the Leu permease LAT-1 cycles between the endosomal compartment and the plasma membrane depending upon amino acid availability, BinA promoted LAT-1 retention at the plasma membrane irrespective of amino acids abundance. Second, we demonstrate that LAT-1 anterograde transport depends on Rab21 activity, just like BinA-dependent TORC1 sensitization. Third, we mapped a central domain of BinA that mediates both TORC1 sensitization and LAT-1 anterograde transport, which indicated that both processes are linked. Collectively, our data is consistent with the mechanism where a central domain of BinA promotes LAT-1 anterograde trafficking through Rab21/Rab22, which in turn facilitates rapid import of exogenous Leu directly at the plasma membrane increasing the intracellular amino acids pool and triggering TORC1 signaling. The revised version now includes two new figures (Figs 11 & 12) and 2 new figure panels in Fig 13.

The following list summarizes the major new experiments included in the revision:

(1) We investigated trafficking of endogenous LAT-1 in A549 cells in response to abundance of exogenous amino acids. Some cells were also treated with phorbol ester which triggers internalization of plasma membrane LAT-1. The data shows that under replete conditions LAT-1 is primarily endosomal but is re-localized to the cell surface upon amino acid withdrawal. The new data is included in Fig11 A-C.

(2) Imaging of A549 cells expressing GFP-Rab21 or GFP-Rab22 was performed to determine if endogenous LAT-1 transverses the recycling endosomal compartment. Indeed, endosomal LAT-1 was found within Rab21 and Rab22+ vesicles after plasma membrane internalization. The new data is included in Fig11 D-F.

(3) Imaging of endogenous LAT-1 cycling from endosomes to the plasma membrane and back in A549 cells expressing either GFP-21 or the GDP-locked dominant negative mutant GFP-Rab21 TN uncovered that Rab21 TN blocked anterograde trafficking in response to exogenous amino acids. The new data is included in Fig11 G-I.

The new data from (1), (2) and (3) demonstrate the LAT-1 cycle from the endosomal compartment to the plasma membrane and back is regulated by exogenous amino acids through the activity of the small GTPase Rab21.

(4) Imaging data of endogenous LAT-1 in cells expressing GFP-BinA demonstrates that BinA triggered LAT-1 anterograde transport resulting in accumulation of surface LAT-1 in the presence of exogenous amino acids. Under the same conditions, LAT-1 remained primarily endosomal in cells expressing GFP or the constitutively active GFP-RagA QL allele. These data demonstrate that BinA regulates LAT-1 trafficking and positioned the permease at the plasma membrane, which facilitates instant access to the pool of exogenous amino acids. The new data is included in Fig 12 A-E.

(5) A series of BinA N-terminal and C-terminal truncation alleles was generated and their capacity to sensitize TORC1 in response to Leu was determined with the canonical starvation-refeeding experiments using phosphorylation of rS6p as a readout. These results lead to identification of a central domain of BinA that mediates TORC1 sensitization. The new data is included in Fig. 13F.

(6) The capacity of the different BinA truncation alleles to trigger LAT-1 anterograde transport was also assessed in A549 cells. The data demonstrates that only alleles that contain the identified TORC1 sensitization domain also triggered LAT-1 anterograde transport. Thus, BinA regulation of TORC1 sensitization and LAT-1 anterograde transport share the same molecular determinants. This new data is included in Fig. 13G.

The following is a point-by-point response to the reviewer’s comments:

**Reviewer #1:**

**Major points**

*“1. The authors showed that BinA enhances amino acid import via the LAT1. LAT1 facilitates the uptake of large neutral amino acids, including Leu and Met. The authors also showed that BinA responds to Leu but not Met. Why does not BinA sensitize mTOR in response to Met while it can likely enhance the intracellular import of Met?”*

**Response**: The reviewer brings an excellent point given that there is a level of substrate promiscuity and functional redundancy in amino acid transporters. Why does BinA sensitize TORC1 to Leu but not Met, even though LAT-1 can transport Met as well? Two factors likely account for the phenotypic difference: **(i)** the affinity of LAT-1 for Leu is higher as compared to Met, which likely produces distinct transport kinetics and efficiency especially at lower substrate concentrations; **(ii)** unlike Met, the intracellular abundance of both Leu and Arg is relayed to TORC1 via a direct sensory mechanism where distinct sensors directly bind Leu or Arg. Conversely, Met is sensed indirectly by the TORC1 pathway through the abundance of its metabolite S-adenosylmethionine (SAM). SAM production from Met depends on the rate limiting enzyme methionine adenosyltransferase (MAT) and importantly SAM also serves as a universal donor in a large number of cellular methylation reactions. Therefore, small changes in Met import might not be immediately reflected in the SAM pool the way small changes in intracellular Leu and Arg abundance are directly relayed to TORC1. This is especially pertinent if SAM is being shunted for methylation.

*“2. Given that the import of Arg and Leu is mediated by different amino acid transporters, does BinA also enhance the uptake of Arg?”*

**Response**: Unfortunately, we were unable to measure Arg uptake in part because we did not have the molecular tools for assessing Arg import. Also, we could not find an anti-CAT-1 antibody that works well for immunofluorescence, which we could potentially use in CAT-1 trafficking experiments.

*“3. The authors showed that the GTP/GDP state of Rab22 and Rab21 is critical to BinA-mediated TOC1 sensitization, however, the authors also showed that the GEF activity of BinA is not required for the sensitization. How does BinA affect the GTP/GDP state of small GTPases? and how does BinA sensitize TORC1?”*

**Response**: BinA has been shown to promote GDP for GTP exchange for Rab5, Rab21 and Rab22 but not Rab1 or Rab6 (PMID: 25821953), i.e. BinA has a GEF activity towards Rab5 family of Rab GTPases. Direct binding of recombinant BinA to recombinant Rab5 was also demonstrated in the same paper. Given that GEFs must directly interact with their cognate GTPase to facilitate nucleotide exchange, BinA also must directly bind Rab21 and Rab22 as well. It is notable that bacterial GEFs that facilitate subversion of host GTPases can have additional activities to prevent host GAPs from turning off the manipulated GTPase. One example is the *Legionella*-encoded T4SS effector DrrA, which is a GEF for Rab1 (PMID: 16906144) but also AMPylates Rab1 (PMID: 20651120) to lock Rab1 in a constitutively active GTP-bound state by preventing host GAPs from deactivating Rab1.

The reviewer asked how does BinA sensitize TORC1? In the revision we provided new data showing that BinA promotes LAT-1 anterograde trafficking from recycling endosomes to the cell surface thus placing LAT-1 within direct access to exogenous amino acids for import. In the original submission we showed that LAT-1 import increased when BinA was present. We also demonstrated that LAT-1 anterograde trafficking depended on Rab21 activity, just like TORC1 sensitization. Importantly, we identified a central domain of BinA that mediates both TORC1 sensitization and LAT-1 anterograde trafficking, thus showing that TORC1 sensitization is mechanistically linked to LAT-1 surface localization. Potentially, the BinA central domain might function by preventing deactivation of GTP-bound Rab21 (and Rab22) by host GAPs to sustain LAT-1 anterograde transport, akin to DrrA. In such a scenario, BinA GEF activity would made functionally redundant by host Rab21 GEFs, which explains why BinA GEF mutants still sensitize TORC1.

*“4. The section title” BinA sensitizes TORC1 signaling by altering endosomal trafficking determinants.” It is not clear by saying altering endosomal trafficking determinants. It needs to be specific what is exactly changed in the presence of BinA.”*

**Response**: As per the reviewer’s suggestion we have changed the section title to ‘*BinA sensitizes the TORC1 response to arginine and leucine though the function of the small GTPases Rab21 and Rab22.*’

*“5. The logic of the paper is extremely obscure and some conclusion sentences are terribly convoluted.*

*For example:*

*Line 43-45: “…through rewiring of processes upstream of mTOR by removing Rab5-dependency and replacing it with a Rab21/Rab22-dependency.” Not clear what this means.*

*Line 341-343: “BinA expression did not increase nor accelerate TORC1 signaling in HK293 cells upon stimulation with different doses of LLoMe (Fig 6), which would be expected if BinA functions downstream of amino acid import.”*

*Line 431-434: “Thus, signaling upstream of TORC1 appears to switch from Rab21/22-dependency to Rab5-dependency contingent upon the presence of BinA or RagA Q66L respectively.””*

**Response**: Respectfully, we disagree with this comment. Neither Reviewer #2 nor Reviewer #3 commented negatively on this aspect of the manuscript. On the contrary, Reviewer #2 praised us on this issue – “***Despite the complexity of the subject matter, the manuscript is clearly written and accessible***.” Nevertheless, we have made some changes to the examples highlighted by the reviewer in the hope that clarity can be further enhanced.

Explanations: t
**(i)** *Line 43-45: “…through rewiring of processes upstream of mTOR by removing Rab5-dependency and replacing it with a Rab21/Rab22-dependency.” Not clear what this means.*

Statement was changed in the revised manuscript to “We uncovered that Lpg0393 lowers the activation threshold for mTOR signaling upon stimulation with arginine or leucine by promoting anterograde trafficking of amino acid permeases through subversion of the small GTPases Rab21 and Rab22.”

**(ii)** *Line 341-343: “BinA expression did not increase nor accelerate TORC1 signaling in HK293 cells upon stimulation with different doses of LLoMe (Fig 6), which would be expected if BinA functions downstream of amino acid import.”*

Leu and LLoMe are sensed by the same intracellular sensor upstream of TORC1 and activate the same signaling cascade. The difference between the two molecules is that Leu is imported in the cell via a dedicated transporter, whereas LLoMe crosses the lysosomal membrane by itself. Therefore, one can predict that if BinA subverts the TORC1 signaling cascade directly, a sensitization phenotype will be observed upon stimulation with both Leu and LLoMe. However, that was not the case – only Leu but not LLoMe treatment sensitized TORC1 when cells expressed BinA indicating that BinA likely directly or indirectly regulates amino acid transport.

**(iii)** *Line 431-434: “Thus, signaling upstream of TORC1 appears to switch from Rab21/22-dependency to Rab5-dependency contingent upon the presence of BinA or RagA Q66L respectively.”*

Statement was changed in the revised manuscript to “…Thus, BinA and RagA Q66L sensitize TORC1 signaling via distinct mechanisms, whereby different Rab5 family members participate as regulatory factors in each case....”

Minor Points:

*“1. The cell line used in this study is HEK293 or HK293?”*

**Response**: Cell line nomenclature consistency has been corrected in the revised manuscript

*“2. Line 439: should be Rag GTPase, not Rag GTPare.”*

**Response**: Corrected in the revised manuscript.

**Reviewer #2:**

**Major points:**

**NONE**

Minor Points:

*“1. Author statement “We found that BinA rewires amino acid transport mechanisms by manipulating Rab5 family GTPase…” (line 60): I believe that this statement is not strictly valid. While the authors were able to demonstrate that BinA manipulates the Rab5-family pathway, they did not provide evidence that this manipulation directly affects Rab proteins. The statement implies a direct interaction with Rab proteins, which has not been experimentally confirmed in this study. I would recommend a more cautious phrasing at this and other relevant points.”*

**Response**: BinA has been shown to promote GDP for GTP exchange for Rab5, Rab21 and Rab22 but not Rab1 or Rab6 (PMID: 25821953), i.e. BinA has a GEF activity towards Rab5 family of Rab GTPases. Direct binding of recombinant BinA to recombinant Rab5 was also demonstrated in the same paper. Given that GEFs must directly interact with their cognate GTPase to facilitate nucleotide exchange, BinA also must directly bind Rab21 and Rab22 as well. Thus, we believe that statement is valid given the previously published data (from PMID: 25821953) and the data from our study.

*“2. author statement „In addition, BinA expression alone did not trigger phosphorylation of either S6K or rS6p (Fig 4A-B) in the absence of Arg/Leu indicating that the TORC1 pathway retains stimulus dependency despite the BinA-dependent sensitization.”: Upon examining Fig. 4A, it appears to me that the expression of BinA leads to an increase in S6K phosphorylation in the absence of exogenous arginine supplementation (compare lane GFP/0uM Arg with lane GFP-BinA/0uM Arg). The band intensities differ markedly. Could it therefore be possible that BinA induces mTORC1 stimulation even without concurrent amino acid supplementation?”*

**Response**: We agree that this could be possible however the phenotype in question was not consistently reproducible (for example compare Fig 4A vs 4B) and TORC1 activation in BinA-producing cells in the absence of amino acids is quite low (judging from phosphor-protein band intensity), thus we could not conclude that this is a physiologically meaningful response.

*“3. line 444, “data not shown”: Please provide data in the supplemental material.”*

**Response**: The data is shown in Supplementary Figure 4

**Reviewer #3:**

**Major points:**

**Comment 1)** *“1. The authors should identify the interacting protein(s) of BinA in host cells. The clear cell biological phenotypes and the notion that BinA function by modulating amino acid transporters have provided the information needed to screen candidates obtained by technologies such as Turbo-ID.”*

**Response**: BinA has been shown to promote GDP for GTP exchange for Rab5, Rab21 and Rab22 but not Rab1 or Rab6 (PMID: 25821953), i.e. BinA has a GEF activity towards Rab5 family of Rab GTPases. Direct binding of recombinant BinA to recombinant Rab5 was also demonstrated in the same paper. Given that GEFs must directly interact with their cognate GTPase to facilitate nucleotide exchange, BinA also must directly bind Rab21 and Rab22 as well. All the data from our study indicates that the mechanism for BinA-mediated TORC1 sensitization is mediated through subversion of Rab21 and Rab22 – two GTPases that interact with BinA (based on published literature PMID: 25821953).

**Comment 2)** *“2. It is necessary to obtain a substitution mutant of KinA defective in its activity and used in a few key experiments such as triggering TORC1 activation upon ectopic expression in Figs. 4 and 5.”*

**Response**: As per the reviewer’s suggestion, we have performed comprehensive mutagenesis analysis to uncover a central domain of BinA that mediates both TORC1 sensitization (Fig 13F) and LAT-1 anterograde transport (Fig 13G). The new data is included in figure 13.

Minor Points:

***NONE***

Thank you for considering our manuscript for publication and we look forward to your decision.

Sincerely yours,

Stanimir Ivanov, Ph.D.

Associate Professor

Department of Microbiology and Immunology

B 2-221

1501 Kings Highway

Shreveport, LA 71103

Ph (318) 675-3209

Stanimir.ivanov@lsuhs.edu
