# Supplementary material for: The Legionella pneumophila type IVb secretion system effector BinA subverts amino acid transport to sensitize TORC1 signaling in macrophages
Source: PLoS Pathog. 2026 Jun 8;22(6):e1012998. doi: 10.1371/journal.ppat.1012998 (PMC13258155; doi:10.1371/journal.ppat.1012998)
Supplement: Peer review history

# Peer review: Decision Letter 0

**DOI:** 10.1371/journal.ppat.1012998.r001

**Authors:** David Skurnik

4 Apr 2025

-->PPATHOGENS-D-25-00475

The Legionella pneumophila type IVb secretion system effector BinA subverts amino acid transport to sensitize TORC1 signaling in macrophages

PLOS Pathogens

Dear Dr. Ivanov,

Thank you for submitting your manuscript to PLOS Pathogens. After careful consideration, we feel that it has merit but does not fully meet PLOS Pathogens's publication criteria as it currently stands. Therefore, we invite you to submit a revised version of the manuscript that addresses the points raised during the review process.

Please submit your revised manuscript within 60 days Jun 03 2025 11:59PM. If you will need more time than this to complete your revisions, please reply to this message or contact the journal office at plospathogens@plos.org. When you're ready to submit your revision, log on to https://www.editorialmanager.com/ppathogens/ and select the 'Submissions Needing Revision' folder to locate your manuscript file.

Please include the following items when submitting your revised manuscript:

* A rebuttal letter that responds to each point raised by the editor and reviewer(s). You should upload this letter as a separate file labeled 'Response to Reviewers'. This file does not need to include responses to any formatting updates and technical items listed in the 'Journal Requirements' section below.

* A marked-up copy of your manuscript that highlights changes made to the original version. You should upload this as a separate file labeled 'Revised Manuscript with Track Changes'.

* An unmarked version of your revised paper without tracked changes. You should upload this as a separate file labeled 'Manuscript'.

If you would like to make changes to your financial disclosure, competing interests statement, or data availability statement, please make these updates within the submission form at the time of resubmission. Guidelines for resubmitting your figure files are available below the reviewer comments at the end of this letter.

We look forward to receiving your revised manuscript.

Kind regards,

Holger Sondermann, Ph.D.

Academic Editor

PLOS Pathogens

David Skurnik

Section Editor

PLOS Pathogens

Sumita Bhaduri-McIntosh

Editor-in-Chief

PLOS Pathogens

orcid.org/0000-0003-2946-9497

Michael Malim

Editor-in-Chief

PLOS Pathogens

orcid.org/0000-0002-7699-2064

**Additional Editor Comments:**

As you will see from the detailed comments, two of the reviewers asked for major revisions including new experiments, while one of the reviewers was quite positive in their feedback. All three reviewers agree that your discovery of BinA as a modulator of the mTORC1 pathway is interesting and novel, but they also point out that the underlying mechanism is still poorly understood. This perceived weakness is significant since BinA's function does not seem to rely on its canonical biochemical activity. Hence, further investigation of BinA's molecular mechanism leading to mTORC1 sensitization appears to be a crucial piece of this study that is missing. I would invite you to address this point, however I would leave it up to you whether you choose a proximity biotinylation approach or genetic screen, as suggested by one reviewer, or other means. Please also take into account all other points raised by the reviewers.

**Journal Requirements:**

1) We ask that a manuscript source file is provided at Revision. Please upload your manuscript file as a .doc, .docx, .rtf or .tex. If you are providing a .tex file, please upload it under the item type u2018LaTeX Source Fileu2019 and leave your .pdf version as the item type u2018Manuscriptu2019.

2) We noticed that you used the phrase 'data not shown' in the manuscript. We do not allow these references, as the PLOS data access policy requires that all data be either published with the manuscript or made available in a publicly accessible database. Please amend the supplementary material to include the referenced data or remove the references.

3) Please upload all main figures as separate Figure files in .tif or .eps format. For more information about how to convert and format your figure files please see our guidelines:

https://journals.plos.org/plospathogens/s/figures

**Reviewers' Comments:**

Reviewer's Responses to Questions

**Part I - Summary**

Please use this section to discuss strengths/weaknesses of study, novelty/significance, general execution and scholarship.

Reviewer #1: The MS by Circu et al reported a novel mechanism of mTOR sensitization by Legionella. The authors identified a Legionella effector Lpg0393 (named BinA by the authors) that sensitizes mTOR activity upon stimulation with amino acids Arg or Leu but not Met or Glu. The authors further revealed that BinA sensitizes TORC1 signaling in a RagGTPase complex-dependent manner. The authors also provided evidence that BinA enhances amino acid import through the L-type amino acid transporter 1, LAT1. Furthermore, the authors showed that the small GTPase Rab21 and Rab22 but not Rab5 mediate TORC1 sensitization by BinA upon Arg/Leu stimulation, however, the GTPase activity of BinA is not required. Overall, the finding of BinA as a modulator of mTOR is quite interesting and novel, however, the follow-up mechanistic studies are often contradictory. The molecular mechanism of BinA is still a mystery with all the experiments performed in this study.

Reviewer #2: The peer-reviewed study describes the modulation of eukaryotic mTORC1 signaling by the Legionella effector BinA during infection with the bacterium Legionella pneumophila.

During the infection of eukaryotic cells, Legionella species utilize a specialized secretion system to release effector proteins, including BinA. In the present study, the authors investigate the functional significance and impact of BinA in the context of Legionella infection. Through cellular infection studies, they demonstrate that BinA primarily enhances the activity of the host cell’s mTORC1 complex. The mTORC1 complex responds to metabolic changes, particularly the availability of free amino acids, and plays a crucial role in lipogenesis, which is essential for membrane lipid biosynthesis. Consequently, BinA appears to promote the growth of the Legionella-containing vacuole by increasing mTORC1-controlled lipogenesis. Mechanistically, BinA may exert its effect by manipulating amino acid transport processes.

Despite the complexity of the subject matter, the manuscript is clearly written and accessible. The authors present a wealth of experimental data that elucidate the role of BinA in the infection process. However, it remains unclear which direct binding partners BinA interacts with in the host cell and through which molecular mechanism it regulates mTORC1 activation. While the involvement of Rab proteins is suggested, it is not evident whether these are directly manipulated by BinA. Nevertheless, I consider the impact of BinA on the infection process to be of significant interest, and given the novelty of its modulation of mTORC1, I believe the study merits publication.

Reviewer #3: The study by Circu et al provides interesting evidence to suggest that the Legionella pneumophila effector Lpg0393 (BinA) targets amino acid transport to sensitize TORC1 signaling. The study started with a laborious but effective screen to identify Legionella effectors capable of modulating the mTOR pathway. The authors used a series of cell biology experiments to show that BinA functions by modulating amino acid transport to increase the uptake certain amino acids. Intriguingly, the effects of BinA is independent of its known GEF activity toward a few endosomal Rab small GTPases, which raises the possibility of additional biochemical activity of BinA. Results of the several phenotypic studies are robust and convincing but the study in its current form lacks the mechanistic basis of these phenotypes. This study should be strengthened by identifying the cellular targets of BinA and a BinA substitution mutant that has lost the activity.

**********

**Part II – Major Issues: Key Experiments Required for Acceptance**

Please use this section to detail the key new experiments or modifications of existing experiments that should be absolutely required to validate study conclusions.

Generally, there should be no more than 3 such required experiments or major modifications for a "Major Revision" recommendation. If more than 3 experiments are necessary to validate the study conclusions, then you are encouraged to recommend "Reject".

Reviewer #1: Major concerns:

1. The authors showed that BinA enhances amino acid import via the LAT1. LAT1 facilitates the uptake of large neutral amino acids, including Leu and Met. The authors also showed that BinA responds to Leu but not Met. Why does not BinA sensitize mTOR in response to Met while it can likely enhance the intracellular import of Met?

2. Given that the import of Arg and Leu is mediated by different amino acid transporters, does BinA also enhance the uptake of Arg?

3. The authors showed that the GTP/GDP state of Rab22 and Rab21 is critical to BinA-mediated TOC1 sensitization, however, the authors also showed that the GEF activity of BinA is not required for the sensitization. How does BinA affect the GTP/GDP state of small GTPases? and how does BinA sensitize TORC1?

4. The section title ” BinA sensitizes TORC1 signaling by altering endosomal trafficking determinants.” It is not clear by saying altering endosomal trafficking determinants. It needs to be specific what is exactly changed in the presence of BinA.

5. The logic of the paper is extremely obscure and some conclusion sentences are terribly convoluted.

For example:

Line 43-45: “…through rewiring of processes upstream of mTOR by removing Rab5-dependency and replacing it with a Rab21/Rab22-dependency.” Not clear what this means.

Line 341-343: “BinA expression did not increase nor accelerate TORC1 signaling in HK293 cells upon stimulation with different doses of LLoMe (Fig 6), which would be expected if BinA functions downstream of amino acid import.”

Line 431-434: “Thus, signaling upstream of TORC1 appears to switch from Rab21/22-dependency to Rab5-dependency contingent upon the presence of BinA or RagA Q66L respectively.”

Reviewer #2: (No Response)

Reviewer #3: 1. The authors should identify the interacting protein(s) of BinA in host cells. The clear cell biological phenotypes and the notion that BinA function by modulating amino acid transporters have provided the information needed to screen candidates obtained by technologies such as Turbo-ID.

2. It is necessary to obtain a substitution mutant of KinA defective in its activity and used in a few key experiments such as triggering TORC1 activation upon ectopic expression in Figs. 4 and 5.

**********

**Part III – Minor Issues: Editorial and Data Presentation Modifications**

Please use this section for editorial suggestions as well as relatively minor modifications of existing data that would enhance clarity.

Reviewer #1: Minors:

1. The cell line used in this study is HEK293 or HK293?

2. Line 439: should be Rag GTPase, not Rag GTPare.

Reviewer #2: Specific comments

- Author statement “We found that BinA rewires amino acid transport mechanisms by manipulating Rab5 family GTPase…” (line 60): I believe that this statement is not strictly valid. While the authors were able to demonstrate that BinA manipulates the Rab5-family pathway, they did not provide evidence that this manipulation directly affects Rab proteins. The statement implies a direct interaction with Rab proteins, which has not been experimentally confirmed in this study. I would recommend a more cautious phrasing at this and other relevant points.

- author statement “In addition, BinA expression alone did not trigger phosphorylation of either S6K or rS6p (Fig 4A-B) in the absence of Arg/Leu indicating that the TORC1 pathway retains stimulus dependency despite the BinA-dependent sensitization.”: Upon examining Fig. 4A, it appears to me that the expression of BinA leads to an increase in S6K phosphorylation in the absence of exogenous arginine supplementation (compare lane GFP/0uM Arg with lane GFP-BinA/0uM Arg). The band intensities differ markedly. Could it therefore be possible that BinA induces mTORC1 stimulation even without concurrent amino acid supplementation?

- line 444, “data not shown”: Please provide data in the supplemental material.

Reviewer #3: (No Response)

**********

PLOS authors have the option to publish the peer review history of their article (what does this mean?). If published, this will include your full peer review and any attached files.

If you choose “no”, your identity will remain anonymous but your review may still be made public.

**Do you want your identity to be public for this peer review?**   For information about this choice, including consent withdrawal, please see our Privacy Policy.

Reviewer #1: No

Reviewer #2: No

Reviewer #3: No

[NOTE: If reviewer comments were submitted as an attachment file, they will be attached to this email and accessible via the submission site. Please log into your account, locate the manuscript record, and check for the action link "View Attachments". If this link does not appear, there are no attachment files.]

**Figure resubmission:**

While revising your submission, please upload your figure files to the Preflight Analysis and Conversion Engine (PACE) digital diagnostic tool, https://pacev2.apexcovantage.com/. PACE helps ensure that figures meet PLOS requirements. To use PACE, you must first register as a user. Registration is free. Then, login and navigate to the UPLOAD tab, where you will find detailed instructions on how to use the tool. If you encounter any issues or have any questions when using PACE, please email PLOS at figures@plos.org. Please note that Supporting Information files do not need this step. If there are other versions of figure files still present in your submission file inventory at resubmission, please replace them with the PACE-processed versions.

**Reproducibility:**

To enhance the reproducibility of your results, we recommend that authors of applicable studies deposit laboratory protocols in protocols.io, where a protocol can be assigned its own identifier (DOI) such that it can be cited independently in the future. Additionally, PLOS ONE offers an option to publish peer-reviewed clinical study protocols. Read more information on sharing protocols at https://plos.org/protocols?utm_medium=editorial-email&utm_source=authorletters&utm_campaign=protocols

# Author comment: Author response to Decision Letter 1

**DOI:** 10.1371/journal.ppat.1012998.r002

29 Apr 2026

Attachment Submitted filename: Response to Reviewers.docx

# Peer review: Decision Letter 1

**DOI:** 10.1371/journal.ppat.1012998.r003

**Authors:** David Skurnik

27 May 2026

Dear Dr. Ivanov,

We are pleased to inform you that your manuscript 'The Legionella pneumophila type IVb secretion system effector BinA subverts amino acid transport to sensitize TORC1 signaling in macrophages' has been provisionally accepted for publication in PLOS Pathogens.

Before your manuscript can be formally accepted you will need to complete some formatting changes, which you will receive in a follow up email. A member of our team will be in touch with a set of requests.

Please note that your manuscript will not be scheduled for publication until you have made the required changes, so a swift response is appreciated.

IMPORTANT: The editorial review process is now complete. PLOS will only permit corrections to spelling, formatting or significant scientific errors from this point onwards. Requests for major changes, or any which affect the scientific understanding of your work, will cause delays to the publication date of your manuscript.

Should you, your institution's press office or the journal office choose to press release your paper, you will automatically be opted out of early publication. We ask that you notify us now if you or your institution is planning to press release the article. All press must be co-ordinated with PLOS.

Thank you again for supporting Open Access publishing; we are looking forward to publishing your work in PLOS Pathogens.

Best regards,

Holger Sondermann, Ph.D.

Academic Editor

PLOS Pathogens

David Skurnik

Section Editor

PLOS Pathogens

Sumita Bhaduri-McIntosh

Editor-in-Chief

PLOS Pathogens

orcid.org/0000-0003-2946-9497

Michael Malim

Editor-in-Chief

PLOS Pathogens

orcid.org/0000-0002-7699-2064

***********************************************************

Reviewer Comments (if any, and for reference):

Reviewer's Responses to Questions

**Part I - Summary**

Please use this section to discuss strengths/weaknesses of study, novelty/significance, general execution and scholarship.

Reviewer #1: The authors thoroughly addressed the critics and I accept the the revised the manuscript.

Reviewer #2: The authors have addressed my comments to my full satisfaction.

Reviewer #3: The authors have addressed some of the concerns by new experiments. A key missing point is that the mutants i suggested to isolate for BinA were substitution mutants defective in sensitizing TORC1. Instead the authors constructed a series of truncation mutants, which are less informative as such mutants can lose activity simply due to incorrect folding. Nevertheless, this paper in its current form has an enormous amount of data which in general support the main conclusions.

**********

**Part II – Major Issues: Key Experiments Required for Acceptance**

Please use this section to detail the key new experiments or modifications of existing experiments that should be absolutely required to validate study conclusions.

Generally, there should be no more than 3 such required experiments or major modifications for a "Major Revision" recommendation. If more than 3 experiments are necessary to validate the study conclusions, then you are encouraged to recommend "Reject".

Reviewer #1: N/A

Reviewer #2: (No Response)

Reviewer #3: none

**********

**Part III – Minor Issues: Editorial and Data Presentation Modifications**

Please use this section for editorial suggestions as well as relatively minor modifications of existing data that would enhance clarity.

Reviewer #1: N/A

Reviewer #2: (No Response)

Reviewer #3: none

**********

PLOS authors have the option to publish the peer review history of their article (what does this mean?). If published, this will include your full peer review and any attached files.

If you choose “no”, your identity will remain anonymous but your review may still be made public.

**Do you want your identity to be public for this peer review?**   For information about this choice, including consent withdrawal, please see our Privacy Policy.

Reviewer #1: No

Reviewer #2: No

Reviewer #3: No

# Editor report: Acceptance letter

**DOI:** 10.1371/journal.ppat.1012998.r004

**Authors:** David Skurnik

Dear Dr. Ivanov,

We are delighted to inform you that your manuscript, "The Legionella pneumophila type IVb secretion system effector BinA subverts amino acid transport to sensitize TORC1 signaling in macrophages," has been formally accepted for publication in PLOS Pathogens.

We have now passed your article onto the PLOS Production Department who will complete the rest of the pre-publication process. All authors will receive a confirmation email upon publication.

The corresponding author will soon be receiving a typeset proof for review, to ensure errors have not been introduced during production. Please review the PDF proof of your manuscript carefully, as this is the last chance to correct any scientific or type-setting errors. Please note that major changes, or those which affect the scientific understanding of the work, will likely cause delays to the publication date of your manuscript. Note: Proofs for Front Matter articles (Pearls, Reviews, Opinions, etc...) are generated on a different schedule and may not be made available as quickly.

Soon after your final files are uploaded, the early version of your manuscript, if you opted to have an early version of your article, will be published online. The date of the early version will be your article's publication date. The final article will be published to the same URL, and all versions of the paper will be accessible to readers.

For Research Articles, you will receive an invoice from PLOS for your publication fee after your manuscript has reached the completed accept phase. If you receive an email requesting payment before acceptance or for any other service, this may be a phishing scheme. Learn how to identify phishing emails and protect your accounts at https://explore.plos.org/phishing.

Thank you again for supporting open-access publishing; we are looking forward to publishing your work in PLOS Pathogens.

Best regards,

Sumita Bhaduri-McIntosh

Editor-in-Chief

PLOS Pathogens

orcid.org/0000-0003-2946-9497

Michael Malim

Editor-in-Chief

PLOS Pathogens

orcid.org/0000-0002-7699-2064
